# The delivery of evidence-based preventive care for older Americans with arthritis

**DOI:** 10.1186/ar3086

**Published:** 2010-07-16

**Authors:** Jeffrey R Curtis, Tarun Arora, Pongthorn Narongroeknawin, Allison Taylor, Clifton O Bingham, Jack Cush, Kenneth G Saag, Monika Safford, Elizabeth Delzell

**Affiliations:** 1 Division of Clinical Immunology and Rheumatology, Department of Medicine, University of Alabama at Birmingham, 510 20th Street South, FOT 805D, Birmingham, AL 35294, USA; 2 Department of Epidemiology, University of Alabama at Birmingham, 1530 3rd Ave So, Birmingham, AL 35294, USA; 3 Division of Rheumatology, Department of Medicine, Johns Hopkins University, 5200 Eastern Ave, Baltimore, MD 21224, USA; 4 Baylor Research Institute, 3434 Live Oak St, Dallas, TX 75204, USA; 5 Division of Preventive Medicine, Department of Medicine, University of Alabama at Birmingham, 1530 3rd Ave So, Birmingham, AL 35294, USA

## Abstract

**Introduction:**

Previous research suggests patients with rheumatoid arthritis (RA) may receive suboptimal care with respect to preventive tests and services. We evaluated the proportion of older Americans with RA, psoriatic arthritis (PsA), and osteoarthritis (OA) receiving these services and the specialty of the providers delivering this care.

**Methods:**

Using data from 1999 to 2006 from the Medicare Chronic Conditions Warehouse, we identified persons age >/= 65 in the national 5% sample. Over the required five-year observation period, we identified tests and services recommended for older adults and the associated healthcare provider. Services of interest included dual energy x-ray absorptiometry (DXA), influenza and pneumococcal vaccination, hyperlipidemia lab testing, mammography and colonoscopy.

**Results:**

After accounting for the sampling fraction, we identified 141,140 RA, 6,300 PsA, and 770,520 OA patients eligible for analysis. Over five years, a majority of RA, PsA, and OA patients were tested for hyperlipidemia (84%, 89% and 87% respectively) and received DXA (69%, 75%, and 52%). Only approximately one-third of arthritis patients received pneumococcal vaccination; 19% to 22% received influenza vaccination each year. Approximately 20% to 35% of arthritis patients never underwent mammography and colonoscopy over five years. Concomitant care from both a rheumatologist and a primary care physician was significantly associated with a greater likelihood of receiving almost all preventive tests and services.

**Conclusions:**

Among older Americans on Medicare, the absolute proportion of persons with arthritis receiving various recommended preventive services and screening tests was substantially less than 100%. Improved co-management between primary care and arthritis physicians may in part improve the delivery of preventive care for arthritis patients, but novel systematic interventions in this area are needed.

## Introduction

Providing preventive care for complex patients with chronic medical problems is a challenging endeavor [1]. Poor quality of care for many chronic conditions such as osteoporosis has been documented [2,3] despite the availability of evidence-based guidelines and clear recommendations for managing these conditions [4,5]. Recent efforts in the United States to promote high quality care have raised awareness of adhering to evidence-based national recommendations. Modest incentives through the Medicare program provide further motivation to provide certain preventive services [6].

Despite these recent trends that encourage high-quality care, previous research suggests that patients with inflammatory arthritis such as rheumatoid arthritis (RA) receive suboptimal preventive services and care for concomitant comorbidities [7]. Disease and treatment-related risk factors for adverse outcomes that are associated with RA and other forms of inflammatory arthritis such as psoriatic arthritis (PsA) make the need for these services even more compelling than for the average person or for individuals with non-inflammatory arthritis such as osteoarthritis (OA). For patients with inflammatory arthritis, biologic medications, non-biologic disease modifying anti-rheumatic drugs (DMARDs), and other treatments that cause immunosuppression (for example, long term glucocorticoid use) are associated with a risk for infection that is increased compared to the general population [8,9] and may be partially mitigated with appropriate vaccination. RA is also recognized as an independent risk factor for osteoporosis and fracture [10], making the need for bone mineral density (BMD) testing using dual energy x-ray absorptiometry (DXA) more compelling. Persons with RA have an increased risk for certain malignancies such as lymphoma although they have a slightly lower risk for breast and colon cancer [11-14]. Rates of cardiovascular events (for example, acute myocardial infarction) [15-17] are higher in RA and PsA populations, and these patients are recognized to need more aggressive cardiovascular risk factor management than the general population [18].

Despite the clear importance of these preventive services and screening tests, identifying which of a patient's physicians should be responsible for providing these is sometimes unclear. Primary care physicians may be most well-versed and accustomed to providing these services, yet arthritis specialists (for example, rheumatologists) may have more frequent contact with some of these patients. Furthermore, some RA medications may adversely affect risk factors for the conditions of interest (for example, glucocorticoids on BMD, biologic medications on lipid profiles [19-24]), and rheumatologists prescribing these may therefore have greater opportunity to consider how these medications impact their patients' various risk factors. Among many possible factors, a lack of co-management between primary care and arthritis specialists, poor between-provider communication about who should be responsible for providing preventive services and tests, and time pressures on office visits to manage complex patients, may result in patients failing to receive recommended care.

In light of the greater-than-average need to provide most evidence-based preventive services and screening tests to patients with RA and PsA, we used national Medicare data to study the proportion of RA, PsA and OA patients receiving recommended preventive care *vis a vis* national recommendations for the general population (Table 1). These recommendations advise that all older patients (irrespective of whether or not they have arthritis) receive the services of interest. We compared RA and PsA patients to OA patients, in part used as an internal control group. We selected OA as a comparator condition in order to understand how patients with inflammatory arthritis compared with a similar group of Medicare-enrollees without inflammatory arthritis-related disease and treatment-associated risk factors for infection, fracture, malignancy, and cardiovascular events. Furthermore, we evaluated the factors associated with receipt of each of the services and tests of interest, including the specialty of the physician providing the service, to understand whether involvement of primary care physicians in the management of comorbidities for arthritis patients was associated with an increased likelihood of patients receiving the recommended preventive tests and services.

**Table 1:** National recommendations for screening tests and immunizations

Agency	Screening or immunization	Interval for repeat testing
CDC	Influenza vaccine for adults age 65 and older	one dose every year in the fall or winter
CDC	Pneumococcal polysaccharide for adults age 65 and older	• one dose if unvaccinated • one-time revaccination at least five years after first dose if given prior to age 65
USPTF	Lipid screening for men age 35 and older	Every five years; less or more often if warranted
USPTF	Lipid screening for women age 45 and older if at increased risk for heart disease	Every five years; less or more often if warranted
USPTF	Breast cancer screening for women age 40 and older	Every one to two years
USPTF	Colorectal cancer screening for adults age 50 to 75 years old	• Annual screening with high-sensitivity FOBT • Sigmoidoscopy every five years, with high-sensitivity FOBT every three years • Screening colonoscopy every 10 years
USPTF	BMD testing - all women age 65 and older	No specific interval recommended
NOF	BMD testing - women age 65 and older, and men age 70 and older	Every two years or more often if warranted

CDC, Center for Disease Control; NOF, National Osteoporosis Foundation; USPTF, US Preventive Services Task Force

## Materials and methods

### Data source and study cohort

We obtained person-specific, longitudinal administrative claims data from the Center for Medicare and Medicaid (CMS) from 1999 to 2006 for a random 5% sample of Medicare enrollees. Use of the data was governed by a Data Use Agreement from CMS and approved by the university institutional review board (IRB), which granted a waiver of informed consent. The CMS files used in the analysis included the Denominator, Inpatient, Outpatient, and Carrier files. Physician specialty is identified on each outpatient claim.

In order to identify persons with RA, PsA, and OA, we required at least two ICD9 codes from physician office-visits for these conditions (714.X, 696.0, 715.X) within a 12-month baseline period using previously described and validated algorithms [7,25,26]. In order to assure that all eligible subjects had five years of follow-up, this baseline year was required to be 1999, 2000 and 2001. This same year was also used to assess other covariates of interest. Following this baseline period, beginning on January 1 of the next calendar year, all individuals were required to have five years of continuous Medicare part A + B, and the last date of observation (relevant for the 2001 cohort) was therefore 31 December 2006. Individuals enrolled in a Medicare Advantage plan were excluded (generally 15% to 20% of Medicare enrollees) because their administrative data is typically incomplete. Each individual meeting ICD9 diagnosis criteria was assigned to a mutually exclusive category in the hierarchy of PsA, RA, and then OA. The amount of overlap between PsA and RA was low; 0.8% of RA patients had a concomitant diagnosis of PsA.

### Outcomes of interest

The primary outcomes of interest were receipt of preventive services of various types including dual-energy x-ray absorptiometry (DXA), influenza and pneumococcal vaccination, mammography, colonoscopy, and tests to assess hyperlipidemia (administrative codes available upon request). Patients were considered to have received DXA, pneumococcal vaccination, colonoscopy, and testing for hyperlipidemia if they received this test or service at least once during the five-year observation period. Mammography and influenza vaccination were evaluated at more frequent intervals. Since the focus of this analysis was on preventive testing and not management of abnormal conditions once recognized, patients were credited with having a test no more than once annually. For each preventive service, coding manuals and literature specific to that service were used by the investigators to identify the relevant ICD-9 and Current Procedural Terminology (CPT) codes for inclusion. Codes were reviewed for appropriateness by a professional medical coder.

### Statistical analysis

Descriptive statistics were used to compare demographics, comorbidities, and health services utilization (for example, number of outpatient physician visits, number of hospitalizations) stratified by type of arthritis. The proportion of individuals with each type of arthritis receiving each service within the five-year follow-up period was shown descriptively. Logistic regression was used to evaluate the relationship between the type of arthritis (RA and PsA referent to OA) and receipt of each of the services of interest. Because mammography and influenza vaccination are recommended more often than once every five years, ordinal regression was used to evaluate mammography (0, 1, >/= 2) and influenza vaccination (yearly) in categories. The proportionality assumption of the ordinal regression was confirmed qualitatively by using multinomial logistic regression with all categories represented as nominal. The potentially confounding variables that we adjusted for conformed to the Aday-Anderson framework [27], which groups these as predisposing factors (for example, age, gender, race), enabling factors (for example, rural/urban residence, geographic region, median household income defined by census block group, receipt of care from a specialist), and need-based (for example, comorbidities, long term care).

The specialty of the physicians providing each service was also identified. Because the primary focus of this analysis was whether primary care physicians or arthritis specialists provided the services of interest, we evaluated the proportion of patients with at least one service of each type provided by a rheumatologist, a primary care physician, both, or neither. For the analysis of the provider specialty, claims with non-specific physician specialties (for example, a multi-group practice) were excluded and reduced the number of persons eligible for analysis by approximately 3.5%. All analyses were conducted using SAS 9.2 (SAS Institute, Cary, NC, USA).

## Results

Characteristics of the individuals with RA, PsA, and OA are shown in Table 2. As expected, more than two-thirds of each of the arthritis cohorts was women, and a majority was white. Approximately one-fourth of RA and PsA patients did not have at least two visits with a primary care physician. In contrast, most care for OA patients was delivered by a primary care physician and not a rheumatologist.

**Table 2:** Descriptive characteristics of older Medicare enrollees with rheumatoid arthritis, psoriatic arthritis, and osteoarthritis

Variable	Rheumatoid arthritis N = 141,140	Psoriatic arthritis N = 6,300	Osteoarthritis N = 770,520
Demographics			
Age			
65 to 69	32.5	39.4	23.9
70 to 74	31.2	31.8	27.1
75 to 84	22.3	18.7	25.4
85+	10.4	8.3	15.0
Gender, %			
Female	76.9	60.0	72.9
Male	23.1	40.0	27.1
Race, %			
Asian	0.8	-	1.1
Black	6.4	-	7.7
Hispanic	1.6	-	2.0
Other	1.2	-	1.0
White	90.0	97.1	88.2
Rural/Urban, %			
Urban Core	64.3	71.4	65.1
Not Urban Core	35.8	25.6	34.9
Geographic Region, %			
Northeast	20.5	26.7	20.9
Midwest	26.1	21.9	24.6
West	14.9	13.3	14.8
South	38.5	38.1	39.6
Income in $, %			
0 to - <30,000	20.0	13.7	22.5
30,000 to <45,000	39.4	34.0	38.3
45,000 to <60,000	22.3	22.2	21.2
60,000 to <75,000	10.5	14.6	9.7
75,000+	7.8	15.6	8.3
Comorbidities, %			
Myocardial infarction	2.5	-	2.5
Heart failure	5.6	3.5	6.4
Cardiovascular disease	4.1	5.4	5.4
Dementia	0.5	-	0.8
Chronic pulmonary disease	14.1	11.8	13.4
Cancer (any)	6.7	7.6	7.6
Hypertension	37.1	42.2	49.0
Osteopenia	0.8	-	0.9
Osteoporosis	9.7	5.1	7.0
Closed hip fracture	0.8	-	0.7
Physician Specialty (≥2 visits), %			
No rheumatology and no primary care	6.5	5.1	18.9
Rheumatology but no primary care	23.9	27.0	4.2
Primary care but no rheumatology	30.9	21.6	68.4
Both Rheumatology and primary care	38.7	46.4	8.5
Physician Visits, n	14.3 (0, 142)	15.2 (2, 64)	12.8 (0, 168)
Number of days of inpatient hospitalization, n	2.2 (0, 365)	1.7 (0, 66)	3.0 (0, 348)
Receipt of any Long Term Care, %	2.3	0.6	3.5

Data shown as % or as mean (range). All data were assessed in the 12-month baseline period before the start of the five-year observation period.

Totals may not sum to exactly 100% due to rounding.

Cells with a "-"were suppressed due to requirements imposed by data use agreement restrictions related to small cell sizes

**Table 3:** Proportion of patients with rheumatoid, psoriatic, and osteoarthritis receiving preventive services during five years of follow-up

	RA N = 141,140	PsA N = 6,300	OA N = 770,520
DXA, % (Women)	69.2	74.6	51.6
DXA, % (Men)	36.7	28.6	10.2
Influenza vaccination, %			
Not vaccinated	17.3	15.6	18.5
Only 1 vaccination	8.9	10.2	10.1
Only 2 vaccinations	11.6	13.0	11.9
Only 3 vaccinations	16.6	14.6	16.8
Only 4 vaccinations	24.0	27.9	22.9
Vaccinated all five years	21.6	18.7	19.8
Pneumococcal vaccination, %	33.0	33.0	29.0
Mammography, % (women only)			
None	29.2	20.1	28.2
Only 1	14.2	12.2	13.6
2 or more	56.6	67.7	58.2
Colonoscopy, %	64.8	70.5	64.8
Hyperlipidemia lab testing, %	83.5	88.9	87.1

Data shown as %

Totals may not sum to exactly 100% due to rounding

Table 3 shows the proportion of patients with each type of arthritis receiving various services. A majority of women received DXA (69.2% with RA, 74.6% with PsA, and 51.6% with OA). However, receipt of most other services, irrespective of the type of arthritis, was substantially less than 100%. For example, only about 20% of arthritis patients received annual influenza vaccination every year for each of the five years of observation. Only one-third of arthritis patients received pneumococcal vaccination at least once. Approximately 20% to 30% of women with arthritis did not receive mammography even once, and one-third of arthritis patients did not undergo colonoscopy.

**Table 4:** Adjusted* association between type of arthritis and receipt of preventive services, referent to osteoarthritis patients

Outcome variable	Rheumatoid arthritis OR (95% CI)	Psoriatic arthritis OR (95% CI)
DXA	1.66(1.55, 1.77)	1.55(1.19, 2.02)
Vaccination		
Influenza**	1.02(0.97, 1.07)	0.88(0.72, 1.07)
Pneumococcal Vaccine	1.11(1.05, 1.19)	1.04(0.82, 1.32)
Cholesterol lab testing	0.56(0.52, 0.61)	0.79(0.53, 1.18)
Cancer Screening Tests		
Mammography (Women Only)**	0.65 (0.60, 0.69)	0.81(0.59, 1.1)
Colonoscopy	0.83 (0.78, 0.88)	0.90(0.7, 1.16)

CI, confidence interval; OR, odds ratio.

Results in each column are referent to patients with osteoarthritis. Each row represents a unique model.

* adjusted for demographic variables (age, gender, race, geographic region, median household income, rural/urban), predisposing conditions (AMI, CHF, peripheral vascular disease, cardiovascular disease, dementia, COPD, peptic ulcer disease, diabetes with and without complications, paraplegia, chronic kidney disease, cancer, severe liver disease, Alzheimers, hypertension, osteopenia, osteoporosis), prior history of fractures (hip, ankle, clavicle, distal radius/ulna, other radius/ulna, carpal bone, spine, tibia-fibula, humerus, femur, pelvis), health services utilization (hospital days, number of physician visits, days in long term care, physician specialty)

** odds ratios obtained using ordinal logistic regression, grouped as (0, 1, >/= 2) for mammography tests, and (0, 1, 2, 3, 4, 5) for number of annual influenza vaccination

Table 4 shows the prevalence odds ratios for each of the preventive services and tests comparing RA and PsA patients to OA patients. As shown, RA and PsA patients were more likely to receive DXA than OA patients. RA patients were somewhat more likely to receive pneumococcal vaccination but less likely to undergo cholesterol testing or cancer screening.

**Table 5:** Factors associated* with preventive tests and services and among RA patients

	DXA	Influenza vaccination	Pneumococcal vaccination	Hyperlipidemia lab testing	Mammography	Colonoscopy
	OR (95% CI)	OR (95% CI)	OR (95% CI)	OR (95% CI)	OR (95% CI)	OR (95% CI)
**Age**						
65 to 69	1.00 (Ref)	1.00 (Ref)	1.00 (Ref)	1.00 (Ref)	1.00 (Ref)	1.00 (Ref)
70 to 74	0.93 (0.81, 1.07)	0.96 (0.87, 1.07)	0.89 (0.78, 1.00)	0.72 (0.60, 0.86)	0.71 (0.61, 0.81)	1.07 (0.94, 1.21)
75 to 79	0.62 (0.54, 0.72)	1.02 (0.91, 1.14)	0.92 (0.80, 1.06)	0.62 (0.51, 0.74)	0.51 (0.44, 0.60)	0.75 (0.65, 0.86)
80 to 84	0.49 (0.40, 0.59)	1.00 (0.86, 1.16)	0.82 (0.68, 0.98)	0.36 (0.29, 0.44)	0.28 (0.23, 0.33)	0.73 (0.61, 0.87)
85+	0.30 (0.22, 0.40)	0.96 (0.76, 1.21)	0.65 (0.48, 0.88)	0.25 (0.19, 0.35)	0.15 (0.11, 0.21)	0.49 (0.38, 0.65)
**Gender**						
Female	1.00 (Ref)	1.00 (Ref)	1.00 (Ref)	1.00 (Ref)		1.00 (Ref)
Male	0.22 (0.19, 0.25)	1.12 (1.01, 1.24)	0.99 (0.88, 1.12)	1.18 (1.00, 1.40)	Women Only	0.99 (0.88, 1.12)
**Race**						
White	1.00 (Ref)	1.00 (Ref)	1.00 (Ref)	1.00 (Ref)	1.00 (Ref)	1.00 (Ref)
Asian	0.67 (0.37, 1.21)	1.11 (0.69, 1.79)	1.12 (0.63, 1.99)	1.52 (0.59, 3.91)	1.05 (0.90, 1.22)	0.75 (0.43, 1.32)
Black	0.58 (0.46, 0.72)	0.41 (0.34, 0.50)	0.95 (0.76, 1.19)	0.83, (0.63, 1.09)	1.06 (0.92, 1.22)	1.21 (0.97, 1.51)
Hispanic	0.74 (0.49, 1.11)	0.48 (0.34, 0.67)	0.68 (0.44, 1.07)	0.97 (0.57, 1.67)	1.12 (0.95, 1.33)	0.73 (0.50, 1.08)
Other	0.70 (0.44, 1.13)	0.51 (0.35, 0.75)	0.60 (0.35, 1.02)	0.67 (0.38, 1.17)	0.99 (0.87, 1.12)	0.83 (0.53, 1.30)
**Income, $**						
0 to <30,000	1.00 (Ref)	1.00 (Ref)	1.00 (Ref)	1.00 (Ref)	1.00 (Ref)	1.00 (Ref)
30,000 to <45,000	1.07 (0.93, 1.24)	1.22 (1.09, 1.38)	1.16 (1.01, 1.34)	1.07 (0.90, 1.28)	0.99 (0.67, 1.49)	1.24 (1.08, 1.43)
45,000 to <60,000	1.43 (1.20, 1.69)	1.52 (1.33, 1.74)	1.19 (1.01, 1.40)	1.40 (1.13, 1.73)	0.61 (0.48, 0.79)	1.35 (1.15, 1.59)
60,000 to <75,000	1.15 (0.94, 1.42)	1.45 (1.22, 1.71)	1.25 (1.02, 1.53)	1.48 (1.12, 1.95)	0.83 (0.60, 1.16)	1.29 (1.06, 1.58)
75,000+	1.28 (1.02, 1.62)	1.57 (1.30, 1.89)	1.28 (1.02, 1.60)	1.57 (1.14, 2.16)	0.92 (0.69, 1.23)	1.70 (1.35, 2.15)
**Physicians providing care (≥2 visits)**						
Rheumatology but no primary care	1.00 (Ref)	1.00 (Ref)	1.00 (Ref)	1.00 (Ref)	1.00 (Ref)	1.00 (Ref)
Both rheumatology and primary care	1.05(0.91, 1.21)	1.71(1.53, 1.91)	1.21(1.05, 1.38)	1.28(1.07, 1.53)	1.41(1.21, 1.63)	1.35(1.18, 1.55)
Primary care and no rheumatology	0.56(0.49, 0.65)	1.32(1.18, 1.48)	0.97(0.84, 1.11)	1.34(1.12, 1.6)	1.03(0.89, 1.2)	1.06(0.92, 1.21)
No rheumatology or primary care	0.66(0.52, 0.83)	0.87(0.72, 1.05)	0.77(0.61, 0.98)	0.87(0.66, 1.13)	1.05(0.83, 1.35)	1.17(0.93, 1.46)

CI: confidence interval; OR: odds ratio

*adjusted for all factors listed for Table 4.

Over the five-year observation period, among RA, PsA, and OA patients who had at least one test or service performed, the proportion who had the test or service provided by a rheumatologist (with or without additional tests or services provided by a primary care physician) was 50.2%, 43.1%, and 17.5% for DXA; 17.7%, 14.5%, and 2.3% for at least one influenza vaccination; 9.6%, 6.9%, and 1.1% for pneumococcal vaccination; and 11.0%, 11.4%, and 1.8% for any hyperlipidemia lab test. Physician specialty was further examined for RA patients in Table 5, which described and controlled for additional factors associated with these services among RA patients (insufficient numbers of PsA patients were available within the data to permit analogous results). As shown, older patients, African Americans, and those with lower incomes were significantly less likely to receive most preventive tests and services. Men were more likely to be tested for hyperlipidemia. Higher income was associated with receipt of all preventive tests and services except for mammography, which varied little across income groups. For DXA, care from a rheumatologist, with or without concomitant care from a primary care physician, was significantly associated with receipt of DXA. In contrast, compared to care provided only by a rheumatologist, RA patients were significantly more likely to receive all other preventive tests and services if they had concomitant care from a primary care physician.

## Discussion

Among older Americans with RA, PsA, and OA our results show that over a five-year observation period, important preventive tests and services such as influenza and pneumococcal vaccination were substantially underutilized. Only 19% to 33% of arthritis patients received these vaccinations as recommended. In contrast, screening for other health-related issues with mammography, colonoscopy, DXA and hyperlipidemia lab testing was better, ranging from 40% to 90%. Except for DXA, rheumatologists provided few of these services; more optimal use of preventive tests and services was associated with concomitant care from both a primary care physician and a rheumatologist. However, about 25% of patients with inflammatory arthritis did not have concomitant care from a primary care physician.

Compared with the general population, influenza vaccination and breast cancer screening rates reported in our study are lower than those reported by National Committee for Quality Assurance (NCQA) using the Health Plan Employer Date and Information Set (HEDIS) data [2]. HEDIS data are annually obtained from administrative claims, medical record review of a random sample of eligible patients, or a combination of both. The influenza vaccination rate from HEDIS in the general population (69%) represents the percentage of adults aged 65 and older who receiving an influenza vaccination during the most recent flu season. The breast cancer screening rate (67%) in HEDIS represents the percentage of women 40 to 69 years who had a mammogram to screen for breast cancer within the last two years. The colonoscopy rates in our cohort are higher than the colorectal cancer screening rate reported in the HEDIS (50%), despite more liberal definitions used by HEDIS which allow for any of the four following tests: fecal occult blood test (FOBT) during the measurement year, flexible sigmoidoscopy during the past five years, double contrast barium enema during the five years, and colonoscopy during the past 10 years.

The proportion of arthritis patients with BMD measurement in our study was higher than previously reported for the general U.S. Medicare population age >/= 65 years; in the general population, only about one-third of women and <5% of men had received BMD testing at any time over a seven-year period [28]. Because many rheumatologists have in-office DXAs and bill for this service [28], they likely are more attuned to providing DXA to their patients. We also found that the performance rates were relatively high for hyperlipidemia screening (83% to 90%) compared with other preventive services. They were similar to the 81% to 88% rates reported by NCQA and others [2]. This may be due to there being fewer barriers to testing and ready accessibility of hyperlipidemia lab testing to physicians of all specialties, in contrast to other services such as DXA and colonoscopy which require access to special equipment or physicians with specialized training in performing this procedure.

Interestingly, starting at approximately age 75, advancing age was associated with a lower likelihood of receipt of DXA, hyperlipidemia lab testing, and cancer screening, despite age clearly being a risk factor for fracture, cardiovascular disease (CVD), and malignancy. This may be related to a physician's and patient's lack of expectation of benefit of these services, perhaps in relation to concern for an offsetting mortality risk from other causes. However, because our analysis intentionally included only individuals who remained alive and under observation for five years, our analysis represents a healthier group of individuals with arthritis. For this reason, the preventive tests and services we studied would seem to be even more appropriate than for a less select population where offsetting mortality risk may attenuate the benefit of screening tests. There are likely additional explanations for why older patients were less likely to receive most preventive tests and services; these reasons might include more limited access to care (potentially affected by arthritis-related disability), and patients' refusal in light of their own goals and values [29].

Focusing particularly on RA, where more comparative literature is available, our findings are consistent with previous population based studies showing generally low preventive health care and screening services delivered to RA patients. In 2000, MacLean *et al*. have raised awareness of the need for increased attention to preventive care for patients with RA [7]. This study assessed quality of various services that RA patients received for their arthritis, comorbid diseases, and health care maintenance by using administrative insurance data over a four-year period (1991 to 1995). The overall quality score for health care maintenance, which included colorectal cancer screening (colonoscopy or barium enema once every five years for persons over 50), breast cancer screening (mammogram annually for women aged 50 to 70), and cervical cancer screening (Papanicolaou testing every three years for women aged 50 to 70) among eligible RA patients was 42% [7]. Recently, Aizer *et al*.reported [30] that over half of patients with RA participating in the Consortium of Rheumatology Researchers of North America (CORRONA) registry had not received BMD testing despite RA being recognized as an independent risk factor for osteoporosis. Using clinical data from a population-based cohort of patients with RA in Rochester with a median follow-up time of 5.4 years, Kremer and colleagues examined the probability of receiving various preventive medical services including influenza vaccination (once a year for persons over 65), pneumococcal vaccination (one time for persons over 65), mammograms (biennially for ages 40 to 49 and annually for those 50 and over), and a lipid profile (once every five years). Complete medical records were reviewed by trained abstractors using a standardized protocol with predefined variables. In this cohort, the proportion of RA patients receiving influenza vaccination, pneumococcal vaccination, mammograms, and lipid screening were 32%, 38%, 68%, and 88%, respectively [31]. Similar to our results showing that only a small minority of patients receive hyperlipidemia lab testing from rheumatologists, a large not-for-profit health system found that only 2% of these lab tests were ordered by a rheumatologist [32]. Outside of the U.S., several additional studies have reported 36% to 81% influenza and 34% to 54% pneumococcal vaccination rates in patients with RA obtained from self-report, patient survey, and/or chart audit, figures which were largely derived from cross-sectional analyses in hospital-based clinic settings [33-39].

In light of gaps in the use of preventive tests and services we identified for arthritis patients, what can be done to ameliorate this problem? A number of strategies to improve quality of care in rheumatology have been proposed and tested within the boundaries of traditional care processes, with mixed results [40]. Simple interventions involving educating providers via continuing medical education (CME) generally do not change physician behavior or practice [41]. More intensive strategies involving audit and feedback and academic detailing have sometimes been more efficacious [42-45], but effect sizes are often small. Our data suggested better co-management between primary care physicians and rheumatologist might in part improve quality of care. This might be facilitated, for example, by having the arthritis specialists' electronic health record (EHR) notes be generated in real-time and made available (either electronically, or via paper) to the primary care physician, either via electronic exchange (EHR, or facsimile) or hand-carried by the patient [32]. These notes could clearly delineate the patients' health maintenance and preventive services needs and propose the provider responsible for ensuring these services are ordered. At the present, however, electronic health records are used by only a minority of physicians, and EHRs are rarely interoperable. Another potential opportunity may lie in better engaging patients in their own care through use of new personal health records (PHR), which enables patients to better document and perhaps be better advocates for their own healthcare. In light of these emerging information technologies and an increasing focus on quality of care for arthritis patients (at least related to the management of arthritis), new strategies need to be designed and tested to optimize preventive care delivery [46]. It is likely that achieving optimal preventive services in these disease populations will require a shift from fragmented, loosely-defined traditional care to system-based inter-disciplinary care of patient populations with better defined provider roles, nurse coordination of care, disease registries, and continuous quality improvement methods [47].

The strengths of our study include evaluation of the entire U.S. Medicare fee-for-service population and thus our results have high generalizability. Unlike many managed care plans with high turnover, patients typically do not disenroll from Medicare, thus allowing us to have a longer period of follow-up (five years, plus a one-year baseline assessment period) than available in most other health plans. Despite these strengths, our results must be interpreted in light of the study design. It is possible that some services such as influenza vaccination were not billed to Medicare and were provided by another agency (for example, a public health department). Patients might also have been offered these services but declined for a variety of reasons in light of their own preferences and values. Another potential reason for a patient declining services is the requirement for a copayment, a hypothesis supported by our finding that patients with higher income are more likely to receive these services, with the notable exception of mammography. Additionally, we recognize that the optimal interval for repeating some tests (for example, DXA) is not well-specified, particularly if a previous test was normal. However, except for colonoscopy, where testing is recommended at least every 10 years, our observation period of five years would seem long enough such that at least one test or service of each type should have been provided.

## Conclusions

Based upon recommendations from national guidelines applicable to the general U.S. population, patients with arthritis generally received less than optimal care with respect to receipt of preventive tests and services. Although RA patients were more likely to receive BMD testing, they were significantly less likely to receive evaluation for hyperlipidemia or screening for malignancy compared to OA patients. Based upon higher rates and risk factors for adverse events (for example, serious infections, fracture, malignancy, and CVD among patients with inflammatory arthritis, the need for the preventive tests and services we studied is generally more compelling for RA and PsA patients than for patients with OA or the general population. Improved co-management between primary care physicians and arthritis specialists is likely to help improve the quality of preventive care for arthritis patients. However, even for patients who had both a rheumatologist and primary care physician, rates of preventive services were less than recommended. New cost-effective, and generalizable interventions to systematically improve the delivery of preventive care are needed, especially for patients with inflammatory arthritis.

## Abbreviations

BMD: bone mineral density; CME: continuing medical education; CMS: the Center for Medicare and Medicaid; CPT: Current Procedural Terminology; CORRONA: Consortium of Rheumatology Researchers of North America; CVD: cardiovascular disease; DXA: dual energy x-ray absorptiometry; EHR: electronic health record; FOBT: fecal occult blood test; HEDIS: Health Plan Employer Date and Information Set; IRB: university institutional review board; NCQA: National Committee for Quality Assurance; OA: osteoarthritis; PHR: personal health records; PsA: psoriatic arthritis; RA: rheumatoid arthritis

## Competing interests

JC received research grants from Merck, Proctor & Gamble, Eli Lilly, Amgen, and Novartis. JC received consulting/honorarium from Roche/Genentech, UCB, CORRONA, Amgen, Eli Lilly, Merck, and Novartis. ED received research grants from Amgen, and did consulting for Amgen. All other authors declare that they have no competing interests.

## Authors' contributions

JC and ED participated in all areas of the manuscript preparation. TA contributed to the statistical analysis and review of the manuscript. All others contributed to the design of the study, and the writing and review of the manuscript. All authors read and approved the final manuscript.
